# In *Caenorhabditis elegans* Nanoparticle-Bio-Interactions Become Transparent: Silica-Nanoparticles Induce Reproductive Senescence

**DOI:** 10.1371/journal.pone.0006622

**Published:** 2009-08-12

**Authors:** Adam Pluskota, Eva Horzowski, Olaf Bossinger, Anna von Mikecz

**Affiliations:** 1 Institut für umweltmedizinische Forschung (IUF) at Heinrich-Heine-University Düsseldorf, Düsseldorf, Germany; 2 Institute of Molecular and Cellular Anatomy, RWTH Aachen University, Aachen, Germany; Instituto de Tecnologia Química e Biológica, Portugal

## Abstract

While expectations and applications of nanotechnologies grow exponentially, little is known about interactions of engineered nanoparticles with multicellular organisms. Here we propose the transparent roundworm *Caenorhabditis elegans* as a simple but anatomically and biologically well defined animal model that allows for whole organism analyses of nanoparticle-bio-interactions. Microscopic techniques showed that fluorescently labelled nanoparticles are efficiently taken up by the worms during feeding, and translocate to primary organs such as epithelial cells of the intestine, as well as secondary organs belonging to the reproductive tract. The life span of nanoparticle-fed *Caenorhabditis elegans* remained unchanged, whereas a reduction of progeny production was observed in silica-nanoparticle exposed worms versus untreated controls. This reduction was accompanied by a significant increase of the ‘bag of worms’ phenotype that is characterized by failed egg-laying and usually occurs in aged wild type worms. Experimental exclusion of developmental defects suggests that silica-nanoparticles induce an age-related degeneration of reproductive organs, and thus set a research platform for both, detailed elucidation of molecular mechanisms and high throughput screening of different nanomaterials by analyses of progeny production.

## Introduction

We currently observe research and development of nanotechnologies on the fast lane. The number of engineered nanoparticles (NPs) and their applications grow at an equal fast pace. NPs are generated from fundamentally different materials such as amorphous silica, cadmium selenide, carbon or polystyrene, just to name a few. A wide range of chemo-synthetic methods results in an even greater variety of NP-properties. Thus, NPs exhibit characteristic qualities including particle size, morphology, composition, surface area, surface chemistry and reactivity in solution [1]. The very feature of nano-sized particles to take on novel properties and functions in comparison to those seen in the bulk scale disclose a manifold of new technical applications. A growing field of nanobiotechnology covers applications such as drug delivery, drug screening, imaging, diagnosis and gene delivery that, taken together, may enable new strategies for treatment and elucidation of the molecular mechanisms of human disease [2]–[4]. For the development of sustained nanotechnologies it is equally important to investigate nano-bio-interactions to get a clearer picture about effects, including putatively adverse consequences, of NPs on human health [5], [6].

But how to study nano-bio-interactions? It is self-explicatory that *in vivo* studies are desirable, while ethical standards make it mandatory to use animal models. Here we propose to employ the nematode *Caenorhabditis elegans* (*C. elegans*) for the investigation of interactions between NPs and living organisms. *C. elegans* is a transparent roundworm of 1 mm length with a simple anatomy consisting of an invariable number of 959 cells (in the adult hermaphrodite) of which 302 cells are neurons [7], [8]. *C. elegans* possesses a defined cell lineage, e.g. the developmental fate of every single cell has been mapped and is generally invariant between individuals. Despite of the small cell number *C. elegans* exhibits complex tissues such as intestine, muscle, hypodermis, gonad, and a fully differentiated nervous system. A hermaphrodite adult stage which lasts 2 to 3 weeks is preceded by four larval stages (L1 to L4) that are completed in approximately 2 days under typical growth conditions. The fact that many basic physiological processes and stress responses are conserved between *C. elegans* and humans enables comparison of molecular mechanisms. *C. elegans* homologues have been identified for 60–80% of human genes [9]. 12 out of 17 known signal transduction pathways and specific epigenetic marks are conserved in *C. elegans* and humans [10]. The relative ease of cultivation and its transparency allow for observation of organismal end points such as life span and progeny production. A well defined nervous system can be analysed by observation of closely related phenotypes including movement, and egg-laying [11]. A prominent egg-laying defect is the bag of worms (BOW) phenotype which is characterized by intracorporal hatching of eggs in the parent animal. Thus, research on this animal model has contributed to important progress in neuroscience, development, signal transduction, cell death, aging, and RNA interference (http://www.wormbook.org).

We recently showed that fluorescently labelled and unlabelled amorphous silica-NPs enter cells in culture, translocate to the cell nucleus and induce unique alterations of nuclear structure and function [12], [13]. Silica-NPs induce formation of nuclear protein aggregation that exactly recapitulates protein composition and biochemical properties of nuclear inclusions which occur in neurodegenerative aggregation diseases such as Huntington's chorea. In cells treated with silica-NPs inhibition of nuclear processes such as replication, and transcription induces a significant reduction of cell proliferation, whereas cell viability remains unaffected [12].

Here, we report that feeding of wild type *C. elegans* with fluorescently labelled NPs results in different, but reproducible translocation patterns according to the nano-materials used. While life span of NP-fed worms remains unchanged, we observe silica-NP-induced premature reproductive senescence and a significant increase of the BOW phenotype. We show that silica-NP-induced BOW is not due to developmental defects of the egg-laying organ, e.g. the vulva, and can be rescued by the anti-convulsant drug ethosuximide. These results suggest that in the animal model *C. elegans* silica-NPs induce premature degeneration of reproductive organs which may involve their innervation.

## Results

**Figure 1:**
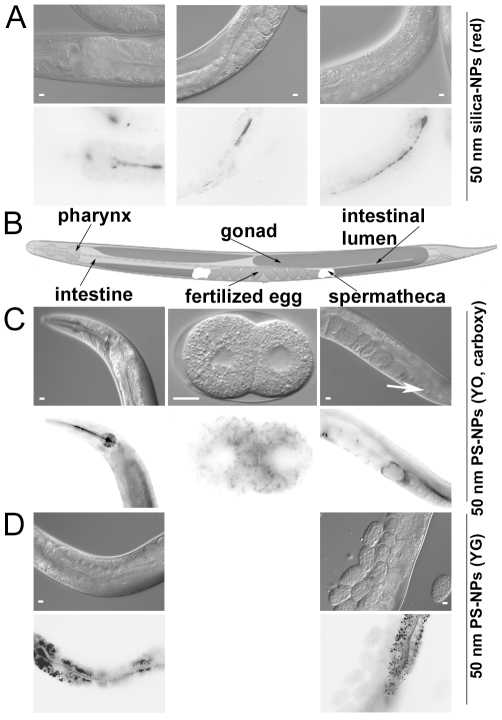
Translocation of fluorescently labelled nanoparticles (NPs) into organs and tissue of the nematode worm *Caenorhabditis elegans*. Young adult hermaphrodites were placed onto agar plates and fed on a bacterial lawn that contained labelled silica-NPs or polystyrene- NPs, respectively. After 16 h of incubation animals were collected and prepared for analysis of nanoparticle distribution by epifluorescence microscopy. Single fluorescence staining was inverted using Adobe Photoshop in order to visualize nanoparticle localization (A, C, D, lower panels). Corresponding nematode anatomy is obtained by differential interference contrast (A, C, D, upper panels). (A) Red-labelled silica-NPs were detectable in the lumen of pharynx and intestine. (B) Schematic representation of *Caenorhabditis elegans* hermaphrodite anatomy. (C) Red (YO, carboxy)-labelled polystyrene-NPs were detectable in the intestine (lumen and tissue), proximal gonad (white arrow), pharynx, spermatheca and cytoplasm of early embryos. (D) Green(YG)-labelled polystyrene-NPs showed a similar translocation to intestine and gonad, but were not detectable in other tissues. Bars, 10 µm.

In order to investigate translocation of NPs, L4 stage larvae or young adult nematodes were placed onto agar plates supplemented with fluorescently labelled silica-NPs (red), red Polystyrene(PS)-NPs (YO, carboxy) or green PS-NPs (YG) and cultivated over night at RT. The comparative experimental design was used based on our previous investigations with silica-NPs in cell culture, and the imaging properties of labelled PS-NPs which due to emission of strong fluorescent signals enable significant uptake studies in cell culture and *in vivo*. Animals were collected, transferred to slides covered with a thin layer of agarose and sealed with a cover slip. Worms were anesthetized with 0.1% Levamisol-solution (Sigma-Aldrich, Schnelldorf, Germany). Red labelled silica-NPs translocated to the lumen of the pharynx and the intestine (Figure 1A, lower panel). Red labelled PS-NPs (YO, carboxy) were enriched in the pharynx, the intestine (lumen and tissue, Figure 1C, lower panel, left), the proximal gonad, and the spermatheca (Figure 1C, lower panel, right, arrow). Detection of PS-NPs in gonads and spermatheca clearly constitutes translocation to secondary organs, whereas silica-NPs were exclusively found in primary organs of entry, e.g. the lumen of the digestive tract. Furthermore, PS-NPs (YO, carboxy) were even detectable in the cytoplasm of early embryos (Figure 1C, lower panel, middle). Green-labelled PS-NPs (YG) were observed in the intestine (lumen and tissue, Figure 1D, lower panel, left) and in lower concentrations in the proximal gonad (Figure 1D, lower panel, right). Ingestion of silica- and PS-NPs clearly follows an uptake-gradient with decreasing concentrations from the anterior to the posterior regions of intestine (Figure S1). Similar uptake patterns were previously demonstrated using bacterial toxins [14] or mercury [15]. Consistent with this our results suggest that NPs are efficiently taken up by *C. elegans* during food intake and translocate to the lumen and tissues of different organs (Table 1). Different translocation patterns may result from imaging properties of respective NPs, since emission of stronger fluorochromes, and respective fluorescent signals is clearly correlated with detection of NPs in “deeper” secondary worm organs. It is important to note, that fluorescence microscopy, as employed here, is limited to a theoretical resolution of 200 nm, and it cannot be excluded that single NPs penetrate further into *C. elegans* tissue or are taken up intracellularly. A more detailed analysis of intracellular translocation of NPs would require establishment of NP-detection in *C. elegans* by electron microscopic techniques. However, to date this has been achieved only by usage of unique nanoparticles, e.g. upconverting nanophosphors that have the property to emit in the visible spectrum in response to IR, visible, UV, and electron irradiation [16]. Despite of these limitations cytoplasmic uptake of PS-NPs (YO, carboxy) was detectable in early embryos (Figure 1C, middle; Table 1).

**Table 1:** Translocation of fluorescently labelled nanoparticles in the model organism *C. elegans* is dependent on their composition.

organ/tissue	silica-NPs	polystyrene-NPs	polystyrene-NPs
	(red)(a)	(YG) (a)	(YO, carboxy) (a)
pharynx - lumen	+(b)	+	+
intestine - lumen	+	+	+
intestine - tissue	**−**	+	+
proximal gonad	**−**	+	+
spermatheca	**−**	**−**	+
early embryo/cytoplasm	**−**	**−**	+

(a) Note that fluorescent signals from polystyrene-NPs are intrinsically stronger versus silica-NPs. YO represents the strongest fluorochrome. It was determined by Fluorescence Correlation Spectroscopy (FCS) that neither of the fluorochromes uncoupled from the respective nanoparticles (Hemmerich and von Mikecz, unpublished results).

(b) Detection of NPs was scored as positive or negative without grading of signal intensity.

Carboxy, carboxyl groups on particle surface; NPs, nanoparticles; YG, yellow-green; YO, yellow-orange.

**Figure 2:**
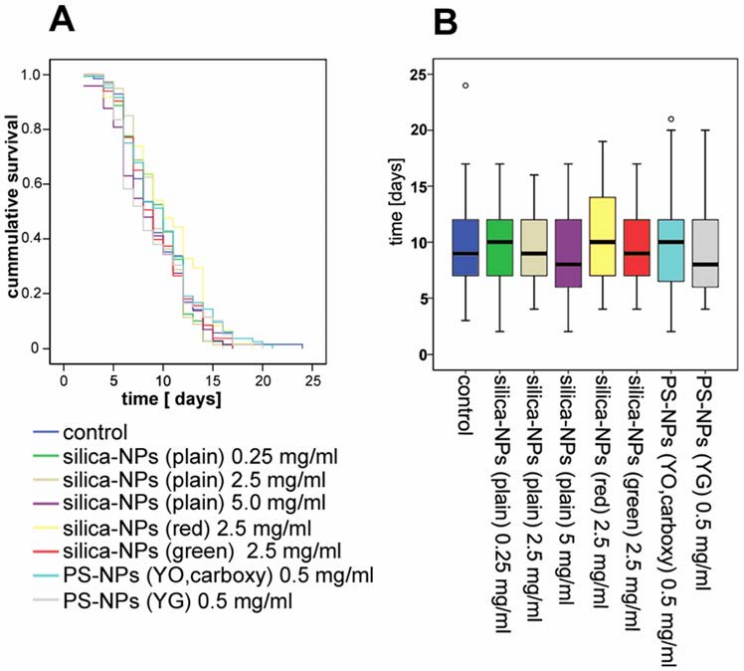
Untreated controls and worms fed on nanoparticles exhibit an identical life span. Worms were cultivated on agar plates overgrown with bacterial lawns that were prepared as indicated. Life span of hermaphrodites was monitored at 25°C as described in Results. (A) Survival curves show no difference in life-expectancy between control (dark blue) and nanoparticle-exposed worms. (B) A corresponding box-plot shows that mean life span of particle-fed worms ranges between 8.6 (±0.45; n = 73) to 10.3 (±0.5; n = 61) days. Untreated nematodes (dark blue) have a mean life span of 9.54 (±0.44; n = 71) days. Analysis by Log-Rank-Test corroborated lack of significant differences in life span. Circles depict two individual worms with abnormally long life spans. Values represent means +/− SD from three independent experiments.

We next investigated life span of *C. elegans* fed on OP 50 *E. coli* (control) or OP 50 supplemented with different NPs. To this end NP-suspensions of indicated particle types and concentrations were applied onto the bacterial lawn of NGM-agar-plates as described above. For control worms the bacterial lawn was supplemented with water. Young adult nematodes were placed onto these plates and transferred to fresh (identically prepared) plates every second day. Worms were grown at 25°C, checked every day and scored for dead animals by absence of reaction upon gentle pokes. Animals that crawled off the plate and worms showing a bag of worms (BOW) phenotype were censored. Each experiment was performed in triplicate and included data of 20 to 40 worms that were analyzed by Kaplan-Meier method (SPSS GmbH Software, München, Germany). Statistical significance was determined by Log-Rank-Test (SPSS GmbH Software, München, Germany). Life span curves of WT worms that were fed on OP 50 (untreated) or OP 50 supplemented with different NPs at concentrations between 0.25 and 5 mg/ml exhibit nearly identical runs of curves (Figure 2A). Mean life span ranged between 8.97 and 10.37 days in NP-exposed *C. elegans*. Control animals had a mean life span of 9.54 days. The corresponding box plot corroborates that there are no significant differences of life span between worms which were fed on different concentrations of plain amorphous silica-NPs, labelled silica-NPs (red), green labelled PS-NPs (YG) or red labelled PS-NPs (YO, carboxy; Figure 2B).

**Figure 3:**
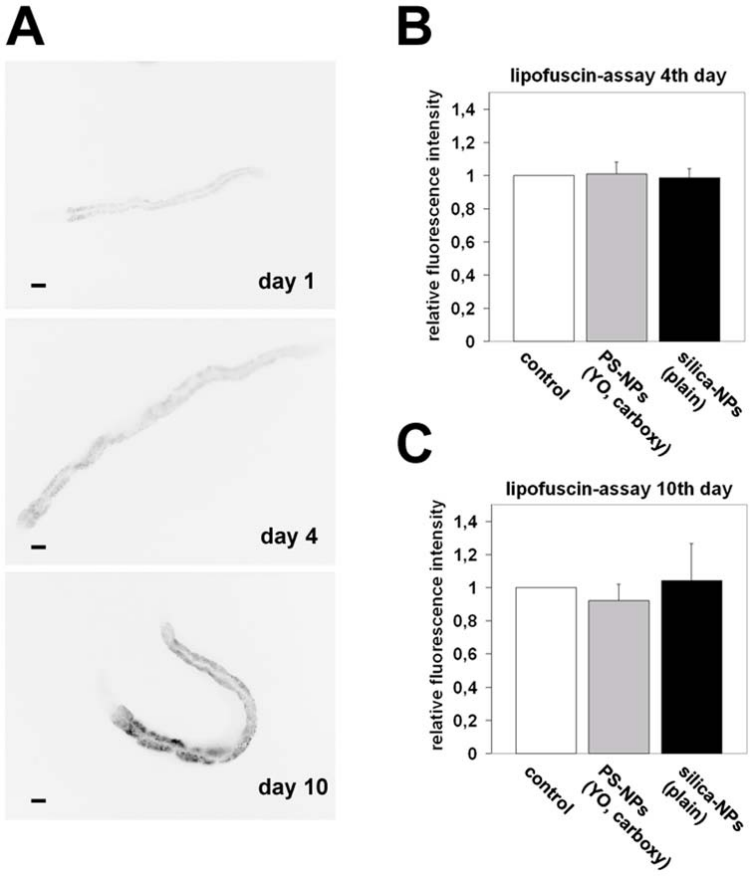
Untreated control worms and *Caenorhabditis elegans* fed on nanoparticles exhibit an identical accumulation of lipofuscin with age. (A) Representative micrographs show increased autofluorescence of lipofuscin in an untreated nematode worm over time. Single fluorescence staining was inverted using Adobe Photoshop. (B, C) Quantitative analysis of fluorescence signals in the intestine from untreated (white) and nanoparticle(NP)-treated worms. Nematodes fed on 0.5 mg/ml polystyrene-NPs (light grey) or 2.5 mg/ml silica-NPs (black), respectively. Specimens were prepared for microscopy and micrographs were acquired at the indicated times. Average fluorescence intensity was measured within regions-of-intrest (ROI) drawn around intestines of each adult hermaphrodite worm. Values represent means +/− SD from three experiments (n = 47, B; n = 30, C). Bars, 50 µm.

In order to validate the life span results formation of lipofuscin was analysed in untreated WT worms and *C. elegans* fed on different NPs. Lipofuscin is an endogenous intralysosomal autofluorescent marker that accumulates in aging *C. elegans* [17]. It is composed of cross-linked protein residues that irreversibly form due to oxidative processes [18]. To monitor the aging process in *C. elegans* NGM-agar-plates with OP50 *E. coli* were prepared as described above with the indicated particle types and concentrations. Synchronized L4 larvae were transferred onto the plates on day 0. Animals were maintained at 15°C. Every second day a fresh NGM-agar-plate (identically prepared) was used as food source. Agarose specimens were mounted at the indicated times and lipofuscin-accumulation measured as described in Materials and Methods. Representative micrographs show that lipofuscin accumulates in the intestine of aging control *C. elegans* from day 1 to day 10 (Figure 3A). Fluorescence quantification revealed that control worms accumulated nearly identical amounts of lipofuscin over time compared with *C. elegans* which were fed on PS-NPs (YO, carboxy) or plain silica-NPs (Figure 3B, C). These results confirm the life span assays (Figure 2) suggesting that nematodes fed with NPs that differ in composition, labelling and functional groups on the particle surface, do not differ from untreated control worms with respect to age-related oxidative processes.

**Figure 4:**
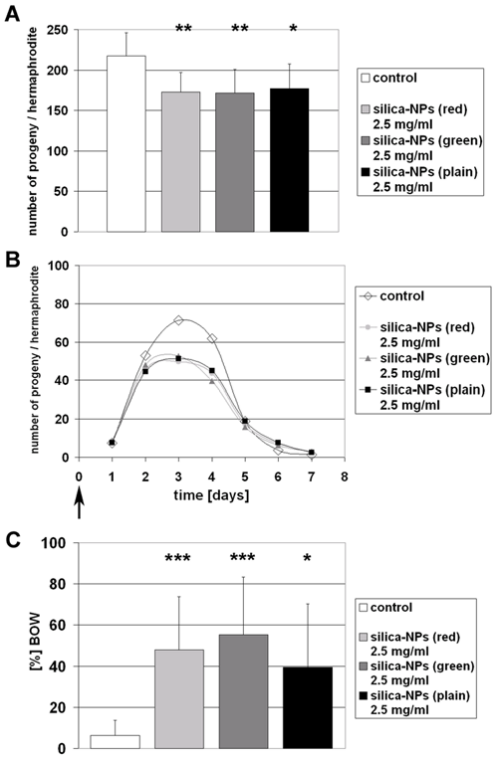
Reproductive life span of *Caenorhabditis elegans* fed on silica-NPs is reduced in comparison to untreated control worms. L4 larvae were placed on OP50 E. coli plates. The bacterial lawn was supplemented as indicated. Adult worms were transferred onto fresh (identically prepared) agar plates daily and residual embryos and larvae were counted as progeny. (A) Progeny production in control (white), and silica-NPs-fed hermaphrodites (light gray, gray, black). (B) Curves show average daily progeny production in control worms and worms fed on silica-NPs. (C) Ratio (%) of the bag of worms (BOW) phenotype that was observed during the progeny production experiments. BOW is significantly elevated in nematodes that fed on silica-NPs. Values represent means (B) +/− SD (A, C) from three experiments (n = 96). *, p<0,05; **, p<0,01; ***, p<0,001.

In cell culture experiments we recently showed that plain and fluorescently labelled silica-NPs penetrate epithelial, fibroblast and neuronal cell types, and significantly alter nuclear structure and function [12], [13]. In cultured cells, silica-NPs induce inhibition of nuclear processes including replication and transcription that results in decrease of proliferation, whereas cell viability remains unchanged. In order to investigate whether silica-NPs affect the progeny production of the model organism *C. elegans* we monitored the reproductive phenotype of untreated versus NP-exposed worms. To this end worms were left untreated or fed on different silica-NPs as described above. On day 0 synchronized L4 larvae were placed on NGM-OP 50-plates with or without NPs. One day later, the adult worms were transferred to fresh, identically prepared, plates. The remaining progeny production (embryos and larvae on the agar) was counted using a Zeiss Stemi 2000 dissecting microscope. This procedure was repeated until the end of the reproductive period, e.g. day 7. Nematodes were maintained at 15°C. Experiments were repeated independently at least four times. The statistical significance between the control and particle-treated groups was determined by one-way ANOVA. Control worms produced on average 217 progeny in seven days, whereas *C. elegans* fed on 2.5 mg/ml plain silica-NPs had significantly less offspring, namely 177 (Figure 4A). Significant reduction of progeny was also observed in *C. elegans* fed on 2.5 mg/ml red-(173 offspring) or green-(172 offspring; Figure 4A) labelled silica-NPs. Analyses with concentrations of plain silica-NPs between 0.25 and 5 mg/ml demonstrated a linear correlation between reduction of progeny and increasing concentrations of NPs (Figure S2). The average daily progeny production as depicted in Figure 4B shows that the timing of the onset of progeny production is not altered by different silica-NPs. However, the levels of steady state progeny production that peaks at day 3 are significantly lower in all NP-fed worms in contrast to the untreated control animals (Figure 4B).

**Figure 5 pone-0006622-g005:**
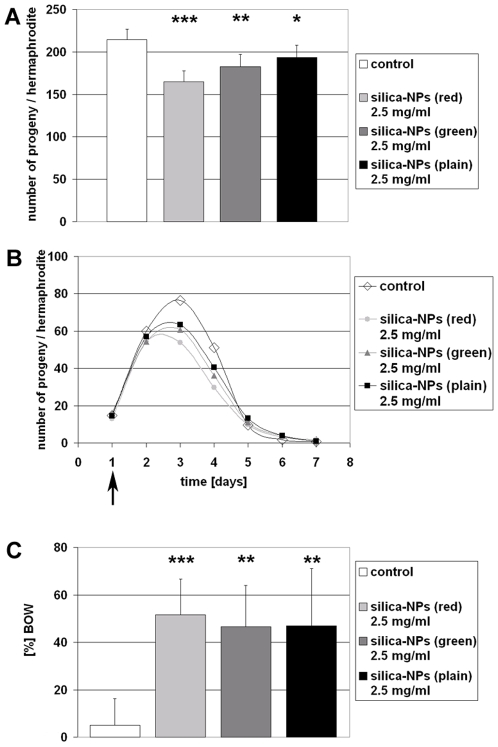
Developmental defects are not responsible for reduced progeny production in *Caenorhabditis elegans* fed on silica-NPs. L4 larvae were placed on OP50 E. coli plates. The bacterial lawn was supplemented as indicated when development into adult hermaphrodite worms was completed (arrow). Adult worms were transferred onto fresh (identically prepared) agar plates daily and residual embryos and larvae were counted as progeny. (A) Progeny production in control (white), and silica-NPs-fed hermaphrodites (light gray, gray, black). (B) Curves show average daily progeny production in control worms and worms fed on silica-NPs. (C) Ratio (%) of the bag-of-worms (BOW) phenotype that was observed during the progeny production experiments. BOW is significantly elevated in nematodes that fed on silica-NPs. Values represent means (B) +/− SD (A, C) from three experiments (n = 60). *, p<0,05; **, p<0,01; ***, p<0,001.

The organ that is critically involved with progeny production at day 3 is the worm's vulva. In the adult *C. elegans* hermaphrodite the vulva constitutes the organ required for egg-laying by connecting the uterus to the external environment. It develops during the postembryonic larval stages L1 to L4 [8], [19]. Defects of vulva development result in impaired egg-laying, and are generally characterized by morphological particularities such as protruding vulva or abnormal eversion of the vulva. To investigate whether NP-treatment impairs vulva development during the last developmental step between L4 and adult, modified progeny experiments were performed by delayed feeding of silica-NPs. In those experiments NP-containing agar-plates were used from day 1, e.g. in adult hermaphrodite *C. elegans* only. Consistent with the idea that developmental defects of parent worms are not involved, we again observed a significant silica-NP-induced reduction of progeny and clear premature cessation of progeny production in those experiments (Figure 5). Morphological inspection of adult hermaphrodites that were fed on silica-NPs corroborated the lack of abnormal vulva phenotypes (data not shown) which had been described previously [19], [20].

However, what could be observed was a significant increase of the BOW phenotype in worms that were fed on plain or labelled silica-NPs (Figure 4C). BOW occurs in 6% of untreated *C. elegans*, and in 39–55% of silica-NP-exposed worms. The BOW phenotype represents an egg laying deficiency in which fertilized embryos hatch from their eggshells within the body of the hermaphrodite and begin to feed upon the tissues of the parent animal. This process normally occurs in aged wild type animals, and in certain mutants where egg-laying is inhibited by defects of vulva development or inadequate motility of the vulval muscles. The latter can be reduced to involvement of intrinsic muscle deficiency or deficient innervation of vulval muscles. By addition of silica-NPs to worms that already completed their development, e.g. adult hermaphrodites, we excluded interference of nanoparticles with vulva development as causative for the BOW phenotype (Figure 5C).

**Figure 6:**
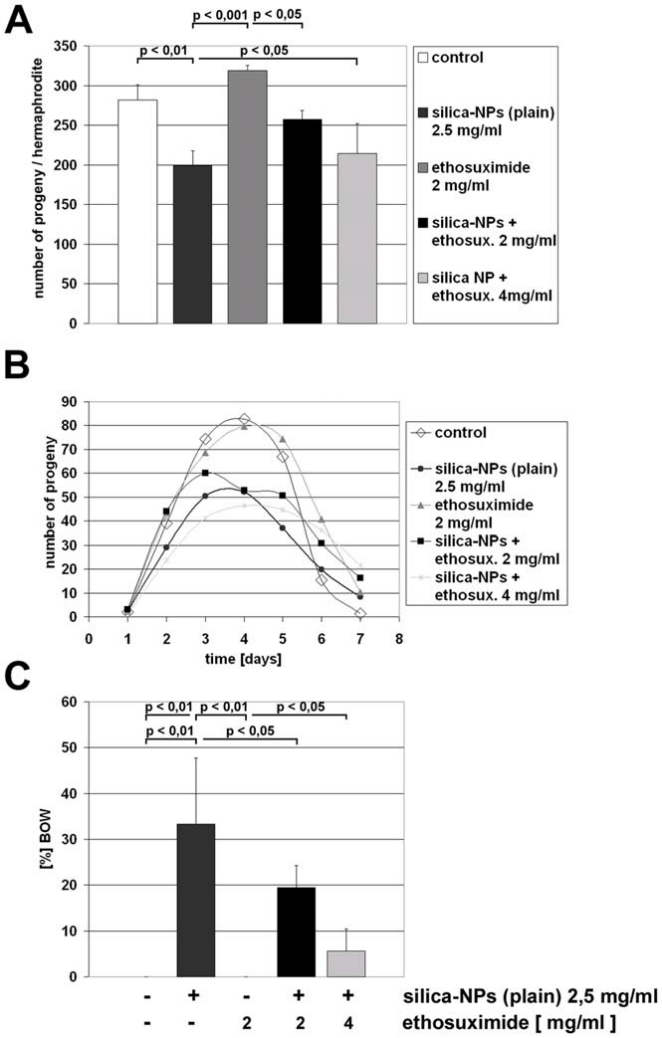
Ethosuximide rescues silica-nanoparticle-induced decrease of progeny production and BOW phenotype. Hermaphrodites were placed onto bacterial lawn on agar at larval stage L4. The bacterial lawn was supplemented as indicated. Adult worms were transferred onto fresh (identically prepared) agar plates daily and residual embryos and larvae were counted as progeny. (A) Progeny production (from left to right) in control (white), silica-NP-fed (anthracite), ethosuximide treated (dark gray) and silica-NP/ethosuximide co-treated worms (black, and light gray). (B) Curves show average daily progeny production in control worms and worms fed with silica-nanoparticles and/or ethosuximide. (C) Ratio (%) of the bag-of-worms (BOW) phenotype that was observed during the progeny production experiments. Values represent means (B) +/− SD (A, C) from three experiments (n = 36). Significance was tested by one-way ANOVA and Tukeys-Post-hoc-test.

In order to investigate the intrinsic functionality of vulval muscle cells the silica-NP-induced BOW phenotype was rescued by addition of the anti-convulsant drug ethosuximide (Figure 6C). It was shown previously that ethosuximide modulates reproductive aging in *C. elegans* [21]. Solid growth medium was pre-treated with either 2 or 4 mg/ml ethosuximide, and nematodes were left untreated or additionally fed with silica-NPs (Figure 6). While silica-NPs induced the expected reduction of progeny, ethosuximide rescued the effect resulting in progeny numbers between those of particle-treated and untreated groups (Figure 6A). Curves that project daily progeny production show that in contrast to all other groups *C. elegans* exposed to ethosuximide and silica-NPs produce more progeny at later time points, e.g. from day four to day seven (Figure 6B). Ameliorative effects of ethosuximide were also observed concerning the BOW phenotype. The mean BOW-ratio of 33.3% in silica-NP-treated hermaphrodites decreased significantly to 5.5% after pre-treatment with 4 mg/ml ethosuximide, and 19.4% after pre-treatment with 2 mg/ml ethosuximide (Figure 6C). The results of the ethosuximide analyses suggest that vulval muscles are not intrinsically defective after treatment of worms with silica-NPs. In fact, ethosuximide may rescue progeny production by modulation of neural function, e.g. vulval innervation [21].

**Figure 7:**
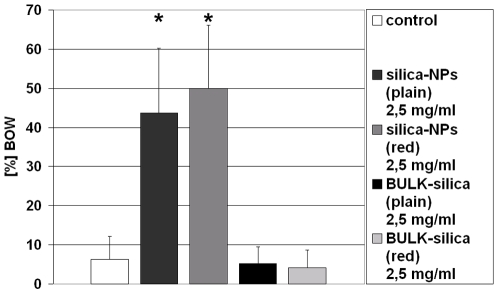
Bulk silica-particles do not induce the BOW phenotype. Hermaphrodites were placed onto bacterial lawn on agar at larval stage L4. The bacterial lawn was supplemented as indicated. Adult worms were transferred onto fresh (identically prepared) agar plates daily for a time period of 7 days. Columns show the ratio (%) of the bag-of-worms (BOW) phenotype that was observed during the indicated treatments. BOW is exclusively elevated in nematodes that fed on silica-nanoparticles. Values represent means +/− SD from eight experiments (n = 96). *, p<0.05.

We next asked whether the silica-NP-induced BOW phenotype represents a nanoparticle-specific biological end point or is likewise inducible by larger silica-particles. To this end *C. elegans* were fed with plain or rhodamine B-labelled silica-particles with a diameter of 500 nm (bulk-silica). While the occurence of BOWs is significantly increased in worms treated with plain and rhodamine B-labelled silica-NPs to 44%, and 51% respectively, bulk-silica do not induce BOWs (Figure 7). The values of plain (5%), and labelled bulk-silica (4%) resemble those of BOW in control animals that were observed as ranging below 10% in all experiments (Figures 4–6C, and 7). Thus, a specific silica-NP property that differs from bulk scale silica seems to account for the BOW egg laying defect, e.g. particle size, surface area or velocity.

## Discussion

We show here that silica-NPs induce a reduction of progeny production in the nematode worm *C. elegans*. This reduction is accompanied by a significant increase of the BOW phenotype which generally occurs in aged wild type worms, and mutants with vulva abnormalities such as complete absence of this egg laying organ or defects of vulval development. Since the latter is completed during the last larval stage (L4), treatment of adult nematodes (Figure 5) excluded vulva development as a target of biological silica-NP effects. In addition, silica-NP-induced BOWs are characterized by a complete oocyte development that ends with hatching of the eggs within the parent's body. Consistent with the idea of an unperturbed oocyte development it has been shown that early mammalian embryonic (oocyte-) development is compatible with fluorescent semiconductor nanocrystal quantum dots [22], and polystyrene-NPs [23]. Rescue of silica-NP-induced BOWs by addition of the anti-convulsant drug ethosuximide rather suggests that impaired neural innervation of vulval muscle cells plays a role in defective egg-laying. In this regard it is important to note that egg-laying in *C. elegans* represents an established phenotype and functional end point of the worm's neural system [11]. A working model of NP-induced impaired innervation matches with previous results obtained in cell culture, where silica-NPs induce protein aggregation that exactly recapitulates neurodegeneration and neurodegenerative disease-associated nuclear inclusions [2], [13]. The emerging concept of NP-mediated interference with neural function has been stimulated by results showing translocation of nano-sized manganese oxide particles to the central nervous system via the olfactory bulb in rodent animal models [24]. At present, it cannot be excluded that silica-NPs induce defective egg-laying in *C. elegans* indirectly, e.g. without translocation of particles to the reproductive organs. However, the observation that bulk silica-particles are unable to induce an increase of BOWs (Figure 7) suggests that distinctive nanoparticle properties are required for impairment of the worm's reproductive system.

Taken together the results presented in this study show that plain and labelled silica-NPs significantly reduce the number of progeny in *C. elegans*. Since aging is defined as degeneration of vital organs, and reproductive aging in *C. elegans* by diminished progeny production [21], we suggest that silica-NPs induce reproductive senescence in these nematodes that is due to degenerative changes in reproductive systems. Accordingly, the frequency of the BOW phenotype that normally occurs in aged wild type worms is significantly elevated by silica-NPs. It is important to note that unlike observations in species such as *Drosophila* and zebrafish the reproductive period, e.g. the onset and cessation of progeny production, cannot be modulated in *C. elegans* by environmental factors. Consistent with this *C. elegans* mutants *daf-2*, *age-1*, and *daf-16* that possess a prolonged life span are not significantly different from wild type animals concerning their reproductive period [25], [26]. Additionally, we ruled out by post larval stage application of NPs (Figure 5) that defects of the development of reproductive organs play a role, and propose that silica-NPs mediate an age-related degeneration of the interaction between neural and reproductive systems.

Here, we suggest progeny production in *C. elegans* as a metric for nano-bio-interactions. Notably, another conventional end point of substance screening in nematode worms, namely analysis of the worm's life span, failed to highlight any specific NP-effects. Similar observations were made in a completely different species showing that *Eisenia veneta* earthworms fed on double-walled nanotubes and C60 fullerenes show abnormalities in reproduction, while survival or mortality remained unchanged [27]. These results fit to the emerging idea that cytotoxicity and organismal death do not represent exclusive biological end points, and should be supplemented by analyses of cellular senescence in cell culture, and reproductive senescence *in vivo*, in animal models. Consistent with this our previous finding that silica-NPs induce cellular senescence in various cell types [12] is validated here by demonstration of reproductive senescence in the animal model *C. elegans*. The observation that different NPs efficiently translocate to distinct *C. elegans* organs and tissues (Table 1) additionally exhibits an ecotoxic aspect, since nematodes of the *Caenorhabditis* species are abundant inhabitants of composts and soils [28], and may thus participate in the food chain. However, the current concentration of engineered NPs in soils is unknown.

Due to its unique characteristics *C. elegans* may be developed into an important model for interactions of multicellular organisms with engineered NPs. Despite of its anatomic simplicity in relation to mammalian animal models and humans *C. elegans* provides a well defined complexity of genes, cells and tissues that can be investigated by means of whole-organism microscopy and high-throughput screening [29]–[31]. Development of high throughput assays for nano-bio-interactions is a necessity in order to keep up with the fast pace of the introduction of new engineered nano-materials and their application. Here, we introduce progeny production as a valuable metric for such analyses. In addition, the conservation of physiological and disease pathways between *C. elegans* and humans provides a particularly suitable research platform to advance our understanding of NP action on human health.

## Materials and Methods

### Worm cultivation

Bristol strain N2 (obtained from the Caenorhabditis Genetic Center stock collection University of Minnesota, St. Paul, MN, USA) was used as wild type (WT) in all experiments. Worms were maintained according to standard procedures that have been described previously [32] on NGM (Nematode-Growth-Medium)-agar-plates (2% (w/v) Agar; 51.3 mM NaCl_2_; 0.25% (w/v) Peptone No. 3; 12.93 µM Cholesterol; 0.5 mM CaCl_2_; 1 mM MgSO_4_×7 H_2_O; 20 mM KH_2_PO_4_; 5.167 mM K_2_HPO_4_) overgrown with *E. coli* (OP 50 strain).

### Nanoparticle treatment

In order to investigate nano-bio-interactions worms were either fed on OP 50, supplemented with deionized water (controls) or NP-suspensions in deionized water in the following concentrations: 0.25 mg/ml or 2.5 mg/ml or 5 mg/ml fluorescently labelled (Rhodamin B, red) and unlabelled (plain) amorphous silica-NPs (50 nm, Kisker, Germany); 0.5 mg/ml (0,05%) fluorescently labelled (yellow-orange, YO) polystyrene(PS)-NPs with carboxyl groups on the surface area (50 nm, Polysciences, Eppelheim, Germany); 0.5 mg/ml (0,05%) fluorescently labelled (yellow-green, YG) polystyrene(PS)-NPs (50 nm, Polysciences, Eppelheim, Germany). 50 µl NP-suspensions were applied to the bacterial lawn with a mean area of approximately 1.76 cm^2^ resulting in an particle-load per area of 142 µg/cm^2^ (5 mg/ml suspension), 71 µg/cm^2^ (2.5 mg/ml suspension), 14.2 µg/cm^2^ (0.5 mg/ml suspension), or 7.1 µg/cm^2^ (0.25 mg/ml suspension), respectively. The dispersity of NPs in suspension was controlled by fluorescence correlation spectroscopy (FCS), showing that single, monodisperse silica and polystyrene NPs constitute the major mobile fraction. Differently sized NP agglomerates did occur, however, in low frequency (Hemmerich and von Mikecz, unpublished results). Where indicated nematodes were fed with 2.5 mg/ml fluorescently labelled (Rhodamin B, red) and unlabelled (plain) bulk silica particles (500 nm, Kisker, Germany) or pretreated with 2 mg/ml or 4 mg/ml ethosuximide (Sigma).

### Microscopy/Fluorescence quantification

Translocation of labelled NPs to *C. elegans* organs or tissue was analysed using a Zeiss Axioplan 2 image microscope (Carl Zeiss MicroImaging GmbH, Göttingen, Germany) with 25×/0.80 Plan-NEOFLUAR or 63×/1.4 Plan-APOCHROMAT objective equipped with a HAMAMATSU Orca II CCD-camera (Hamamatsu Photonics Deutschland GmbH, Herrsching, Germany). For fluorescence pictures a Zeiss Filterset 10 (Exitation 450–490 nm, Emission 515–565 nm) or 15 (Exitation 546 nm, Emission 590 nm) was used. Lipofuscin-accumulation was analyzed using a Zeiss Axioplan 2 image microscope with an 10×/0.3 Plan-NEOFLUAR objective and suitable filter set. Micrographs were acquired by means of Openlab 5.5.0 software (Improvision Deutschland, Tübingen, Germany), and used for quantitative analysis. Regions of interest (ROI) were drawn around lipofuscin fluorescence signals in the worm's intestine and the average fluorescence intensity for each ROI was determined with MetaMorph 4.3 software (Molecular Devices Corp., CA, USA). Further analysis was performed in MS Excel. Statistical significance between treated and untreated groups was measured by one-way analysis of variance (ANOVA) or Tukeys-HSD-Post-Hoc-Test where indicated.

## Supporting Information

Figure S1 Intestinal uptake of fluorescently labelled polystyrene-nanoparticles. Young adult hermaphrodites were placed onto agar plates and fed on a bacterial lawn that contained yellow-green (YG)-labelled polystyrene-nanoparticles (NPs). Fluorescence microscopy indicates high concentration of YG-polystyrene-NPs in the pharynx, and decreasing concentrations from the anterior (left) to the posterior (right) part of the worm (upper micrograph). Corresponding nematode anatomy is visualized by differential interference contrast (lower micrograph). Bar, 50 µm.
(0.99 MB TIF)

Figure S2 A linear correlation between reduction of progeny production and increasing concentration of silica-NPs. Hermaphrodites were placed onto bacterial lawn on agar at larval stage L4. The bacterial lawn was supplemented as indicated. Adult worms were transferred onto fresh (identically prepared) agar plates daily and residual embryos and larvae were counted as progeny. Values represent means +/− SD from four experiments (n = 48). *, p<0,05; **, p<0,01.
(1.71 MB TIF)
