# Supplementary figures and images for: In Caenorhabditis elegans Nanoparticle-Bio-Interactions Become Transparent: Silica-Nanoparticles Induce Reproductive Senescence
Source: PLoS One. 2009 Aug 12;4(8):e6622. doi: 10.1371/journal.pone.0006622 (PMC2719910; doi:10.1371/journal.pone.0006622)

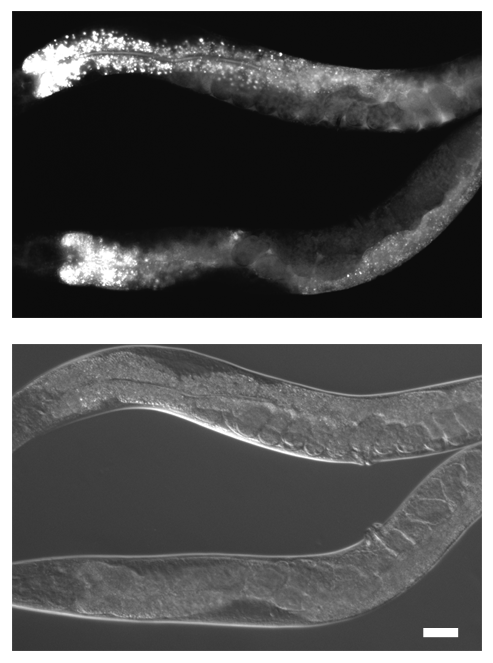

Supplement: Figure S1 — Intestinal uptake of fluorescently labelled polystyrene-nanoparticles. Young adult hermaphrodites were placed onto agar plates and fed on a bacterial lawn that contained yellow-green (YG)-labelled polystyrene-nanoparticles (NPs). Fluorescence microscopy indicates high concentration of YG-polystyrene-NPs in the pharynx, and decreasing concentrations from the anterior (left) to the posterior (right) part of the worm (upper micrograph). Corresponding nematode anatomy is visualized by differential interference contrast (lower micrograph). Bar, 50 µm. (0.99 MB TIF) [file pone.0006622.s001.tif]

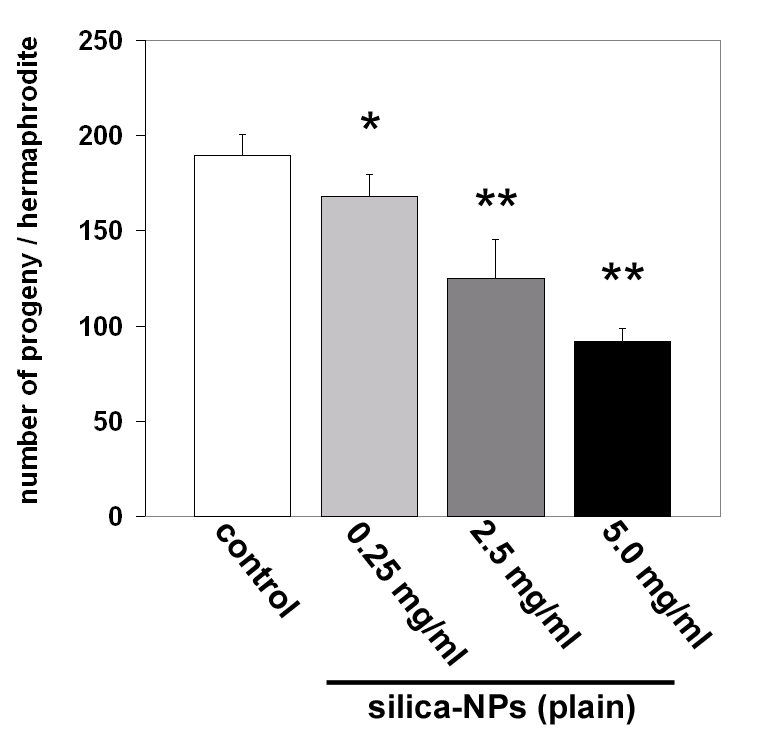

Supplement: Figure S2 — A linear correlation between reduction of progeny production and increasing concentration of silica-NPs. Hermaphrodites were placed onto bacterial lawn on agar at larval stage L4. The bacterial lawn was supplemented as indicated. Adult worms were transferred onto fresh (identically prepared) agar plates daily and residual embryos and larvae were counted as progeny. Values represent means +/− SD from four experiments (n = 48). *, p<0,05; **, p<0,01. (1.71 MB TIF) [file pone.0006622.s002.tif]
